# Biophysical and Biochemical Cues of Biomaterials Guide Mesenchymal Stem Cell Behaviors

**DOI:** 10.3389/fcell.2021.640388

**Published:** 2021-03-25

**Authors:** Jianjun Li, Yufan Liu, Yijie Zhang, Bin Yao, Enhejirigala, Zhao Li, Wei Song, Yuzhen Wang, Xianlan Duan, Xingyu Yuan, Xiaobing Fu, Sha Huang

**Affiliations:** ^1^Research Center for Tissue Repair and Regeneration, Medical Innovation Research Department and the Fourth Medical Center, Chinese PLA General Hospital, PLA Medical College, Beijing, China; ^2^PLA Key Laboratory of Tissue Repair and Regenerative Medicine and Beijing Key Research Laboratory of Skin Injury, Repair and Regeneration, Chinese PLA General Hospital, PLA Medical College, Beijing, China; ^3^Department of General Surgery, The Sixth Medical Center, Chinese PLA General Hospital, Beijing, China; ^4^The Shenzhen Key Laboratory of Health Sciences and Technology, Graduate School at Shenzhen, Tsinghua University, Shenzhen, China; ^5^College of Graduate, Tianjin Medical University, Tianjin, China; ^6^Institute of Basic Medical Research, Inner Mongolia Medical University, Hohhot, China; ^7^Department of Burn and Plastic Surgery, Air Force Hospital of Chinese PLA Central Theater Command, Datong, China; ^8^School of Medicine, Nankai University, Tianjin, China; ^9^Research Unit of Trauma Care, Tissue Repair and Regeneration, Chinese Academy of Medical Sciences, Beijing, China

**Keywords:** microenvironment, biomaterial, stem cell therapies, cell behavior, tissue engineering

## Abstract

Mesenchymal stem cells (MSCs) have been widely used in the fields of tissue engineering and regenerative medicine due to their self-renewal capabilities and multipotential differentiation assurance. However, capitalizing on specific factors to precisely guide MSC behaviors is the cornerstone of biomedical applications. Fortunately, several key biophysical and biochemical cues of biomaterials that can synergistically regulate cell behavior have paved the way for the development of cell-instructive biomaterials that serve as delivery vehicles for promoting MSC application prospects. Therefore, the identification of these cues in guiding MSC behavior, including cell migration, proliferation, and differentiation, may be of particular importance for better clinical performance. This review focuses on providing a comprehensive and systematic understanding of biophysical and biochemical cues, as well as the strategic engineering of these signals in current scaffold designs, and we believe that integrating biophysical and biochemical cues in next-generation biomaterials would potentially help functionally regulate MSCs for diverse applications in regenerative medicine and cell therapy in the future.

## Introduction

Mesenchymal stem cells (MSCs) have received increasing attention in the field of regenerative medicine and tissue engineering due to their high self-renewal ability and multipotential differentiation lineage, as well as accessibility. Numerous studies have shown that they have been applied in repairing cartilage, bone, adipose, muscle, skin, liver, nerve, and other organs (Han et al., 2019). For instance, Wingate et al. (2012) obtained endothelial and muscle-like cells by culturing MSCs on a three-dimensional (3D) hydrogel; Duarte Campos et al. (2015) can accurately guide MSCs toward osteogenic and adipogenic differentiation lineage. Those researches both utilized MSCs as seed cells to successfully harvest corresponding tissue cells, which will provide important cell sources and functional support for subsequent clinical treatment.

However, MSC-based tissue engineering still has its own shortcomings that cannot be ignored. One of the problems is that only a few injected MSCs can home and stabilize on the target tissue and play a therapeutic role (De Becker and Riet, 2016). More important, the uncertainty of the differentiation of MSCs after infusion complicates functional reconstruction. Currently, more and more studies documented that biomaterials could be used to protect transplanted MSCs, especially to maintain the viability of MSCs and accurately induce the MSC to differentiate into specific targeted cells. These potentials are mainly attributed to the ability of biomaterials to mimic a multitude and systematic extracellular milieu to guide MSCs in tissue regeneration. For example, engineered scaffolds derived from different biomaterials lead to the satisfying outcome of MSC-based repair in cartilage regeneration due to the excellent biocompatibility and chondrogenesis induction (Le et al., 2020). In addition, Li et al. (2005) fabricated a biodegradable synthetic scaffold that provides an important microenvironment for MSCs in cartilage repair. Subsequently, the cartilage formation was successfully observed on the scaffold, which provided a practical MSC-based tissue engineering approach for cartilage repair. In addition to the cartilage, Prabhakaran et al. (2009) fabricated a nanofibrous scaffold by electrospinning and successfully observed MSC-derived neural morphology and functional cells for nerve repair. Either as guidance cues or as delivery vehicles for controlling the fate of transplanted cells, biomaterials play a major role in the development of MSC-based therapy.

**FIGURE 1:**
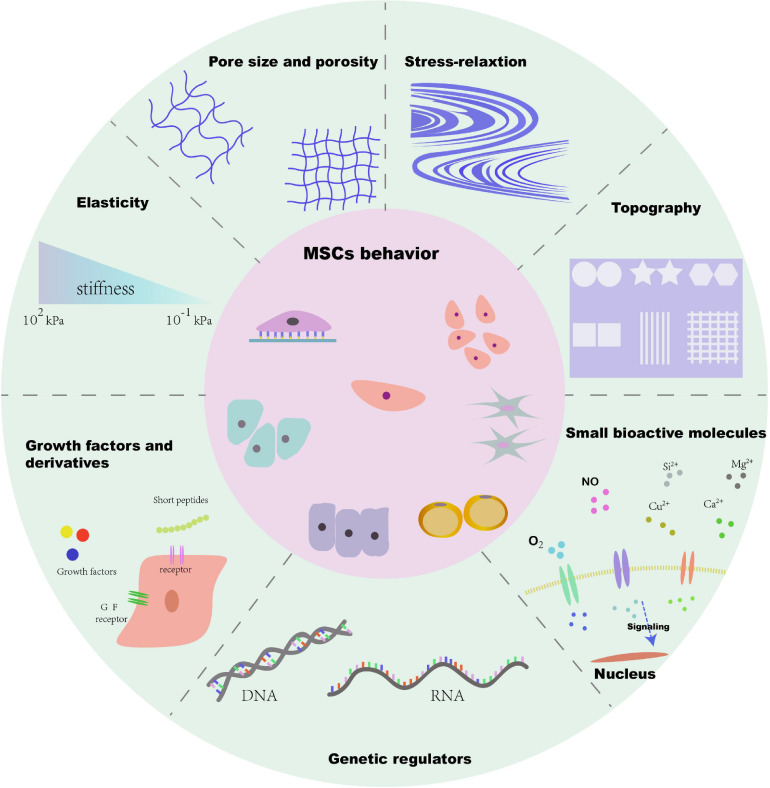
Schematic diagram of biophysical and biochemical cues of microenvironment for MSC behavior.

There is no doubt that numerous factors of biomaterials such as the cellular microenvironment can alter MSC behaviors. These factors include biophysical cues (e.g., stiffness, pore size, porosity, and topography) and biochemical cues (e.g., growth factors, growth factor derivatives, small bioactive molecules, and genetic regulators). Many biochemical cues have been determined over the past century, and a number of studies have reported that biochemical stimulation delivered by biomaterials can influence stem cell attachment, proliferation, and differentiation, and is generally effective and easy to deliver. However, biophysical cues have a longer lifetime and can be easily well defined. For instance, Gilbert et al. (2010) have demonstrated that stiffness of substrate has a significant effect on the fate of muscle stem cell. They elucidated that the soft hydrogel substrate mimics physiological elasticity and can obviously promote the propagation of muscle stem cell, and after transplantation, it contributed extensively to muscle regeneration. Gupte et al. (2018) demonstrated that the chondrogenesis and endochondral ossification of bone marrow stromal cells can be modulated by scaffold pore architecture, specifically pore size. Meanwhile, they found that the pore interconnectivity is essential for capillary ingrowth during bone formation. Thus, MSC-based tissue engineering will be greatly enhanced by the biomaterial design. However, the different designs in a variety of studies have led to difficulties in obtaining clear conclusions about the effects of the different cues and on regulating MSC behaviors. Therefore, it is necessary to focus on summarizing these biophysical and biochemical factors and their significant effects. An overall summary of topics covered in this review is presented in Figure 1. We propose that a deep understanding of various factors of biomaterials is of great significance to better release the potential of MSCs in tissue engineering, and the precise integration of cues according to the needs of different target tissues can open up new avenue for future MSC-based therapy.

## Biophysical Cues

Compared to biochemical cues of biomaterials, biophysical cues have a longer lifetime and can be easily defined. Biophysical cues, such as stiffness, pore size, porosity, topography, as well as stress relaxation, are often regarded as primary elements in biomaterial design for tissue engineering.

### Stiffness

It is well known that, as Discher et al. (2005) claimed, cells can sensitively feel and respond to the stiffness of extracellular matrix. Similarly, the behavior of MSCs can also be altered by stiffness. Engler et al. (2006) cultured naive human MSCs (hMSCs) on matrix with three different levels of stiffness (0.1–1, 8–17, and 25–40 kPa). Finally, hMSCs cultured on the soft substrate, mimicking the brain stiffness at a level about 0.1–1 kPa, exhibited extensive branching and are filopodia-rich, much like primitive neuronal morphology. Stiffer materials that simulate muscle (8–17 kPa) and collagenous bone (25–40 kPa) guided hMSCs exhibiting myoblast-like and osteoblast-like morphology, causing cells to express myogenic marker (MyoD) and osteogenic marker (Runx-2), respectively. Meanwhile, different levels of stiffness can also produce changes in hMSC proliferation. Whitehead et al. seeded hMSCs on the surfaces of common cell culture dishes and polyethylene glycol (PEG)-based hydrogel platform, which have two different levels of stiffness (8–10 and 50–60 kPa). Ultimately, the stem cells on soft hydrogels exhibited more proliferation activity compared to stiffer hydrogels (Whitehead et al., 2018). Stiffness in 3D matrix also has a significant impact on behavior of MSCs. Wingate et al. (2012) synthesized a 3D nanofiber hydrogel matrix with tunable stiffness by varying the time of photopolymerization and stated that hMSCs on the rigid matrices (8–15 kPa) showed a larger extension area and more polarization compared to the soft matrices (2–5 kPa). Meanwhile, the smooth muscle marker (SMA) and SMA mRNA were significantly upregulated. By contrast, the expression of vascular-specific marker (FLK-1) and FLK-1 mRNA was significantly increased on soft substrate. Those results demonstrated that the stiffness of matrix can functionally regulate the behavior of MSCs, and the cells tend to exhibit corresponding morphology, proliferation rate, and differentiation lineages when they are cultured on substrate with similar stiffness to their native niches.

The mechanism of microenvironment affecting the behavior of MSCs mainly focuses on the direction of differentiation. Numerous studies have proposed that stiffness of matrices can modulate self-renewal and differentiation of MSCs via the integrin-mediated signal pathways. Integrins are a family of heterodimeric surface molecules and the most important triggers located in the starting position of mechanotransduction (Du et al., 2011; Higuchi et al., 2013; Sun et al., 2018). For example, Shih et al. (2011) proved that activation of the α2-integrin-mediated Rho kinase (ROCK)–focal adhesion kinase (FAK)–ERK1/2 mechanotransduction pathway could significantly enhance osteogenic differentiation of MSCs, by cultured MSCs on hydrogels with different stiffness. Similarly, Du et al. demonstrated that β1-integrin activation was significantly enhanced in MSCs on soft hydrogels (0.1–1 kPa) compared to hard substrates (50–100 kPa). In addition, soft culture substrates can induce neural differentiation of MSCs by inhibiting the β1 integrin-modulated bone morphogenetic protein (BMP)–Smad pathway (Du et al., 2011). Currently, experts believe that the stiffness of substrate could provide different mechanical stimuli changes in focal adhesion protein (FAP) activities and remolding, and then MSCs sense the changes via integrins, the primary kind of FAP on cell membrane.

### Stress Relaxation

Recently, increasing evidences have shown that stress relaxation of substrate is also a significant mechanical parameter in guiding the behavior of MSCs. Previous researches have mainly focused on the stiffness, which represents the characteristics of purely elastic biomaterials, while most ECMs and tissues are viscoelastic. During the compression process, viscoelastic tissues, such as brain, muscles, and cartilage, require a constantly decreased stress to maintain a certain strain. In other words, native ECM and tissues that maintain constant strain will exhibit stress relaxation or time-dependent deformation, which is another important mechanical property of biomaterials (Chaudhuri et al., 2015; Chaudhuri et al., 2016; Bauer et al., 2017).

Some excellent articles have elucidated the influence of stress relaxation on the behavior of MSCs. Chaudhuri et al. (2016) cultured murine MSC in the hydrogel with tunable stress relaxation but similar initial elastic modulus and found that the faster stress relaxation substrate strikingly enhanced both spreading and proliferation of MSCs compared to the low relaxation hydrogel. In addition, an interconnected, mineralized, and collagen-1-rich matrix was detected in the rapidly relaxing gel, which indicated that the rapid stress relaxation enhanced not only the osteogenesis of MSCs but also the bone-forming activity. In another study, Cameron et al. proposed a polyacrylamide gel system with constant storage moduli but varying loss moduli. The varying loss moduli ultimately changed the creep and deformation of the gel, which represented the stress relaxation property (Cameron et al., 2011). Eventually, they found that the spreading area of MSCs increased with the augmentation in modulus loss and stress relaxation of the substrate. Meanwhile, there was an obvious trend that the proliferation of MSCs was increased on the higher stress relaxation substrate. Furthermore, the differentiation potential of MSCs, such as adipogenesis, osteogenesis, and myogenesis, was also amplified on the higher loss moduli substrates compared to the low loss moduli substrates. Besides, in the calvarial defect repair test *in vivo*, Darnell et al. (2017) found that the rapid-relaxing hydrogel carrying MSCs promoted new bone formation better than the slow-relaxing but stiffness-matched hydrogel. Overall, those studies have shown that the stress relaxation or creep of the substrate can significantly affect the behavior of MSCs in tissue engineering.

With the deepening of the researches, the driving factors behind stress relaxation are gradually being exposed. Although the effects of matrix stiffness and stress relaxation on MSCs are all related to cell focal adhesion and the tension generated by the cytoskeleton or tension fiber, the molecular mechanisms and signal pathways behind them are not completely the same due to the different inherent physical properties. Cameron et al. (2014) demonstrated that the activated mechanotransductive signaling molecules (Rac1) pathway on rapid creep substrates exerted a valid mechanism, helping to the enhance the differentiation of hMSCs toward smooth muscle cell lineage. Chaudhuri et al. (2016) suggested that in the rapidly relaxing gel, the increased binding and clustering of integrins as well as the cell mechanoreceptor actomyosin contractility contributed to the osteogenesis of hMSCs. Compared with the mechanism of stiffness on MSCs, the mechanism of stress relation on cells is different, but there are some overlaps. Therefore, more researches are needed to explore the specific mechanism of stress release.

### Topography

With the progress of the material manufacturing technology and the improvement of resolution, more and more biomaterials with different micro- and nano-patterned surfaces have been manufactured. To explore the influence of terrain on MSCs, various shapes and morphologies, such as squares, stars, stripes, and grids, have been reported (Tang et al., 2010; Tay et al., 2010; Wan et al., 2010). Meanwhile, there is increasing evidence that shows that the geometric characteristics or the topography of the extracellular microenvironment can directly alter the response of MSCs from proliferation, extension, differentiation, and paracrine.

The cell shapes, spreading area, cell–cell interaction, and orientation of arrangement direction are the main targets for biomaterial topography to influence MSCs. Due to the domestication of the surrounding topographical features, MSCs finally adapt to the micro- and nano-patterned surfaces by changing its own morphology, thus changing its own destiny. McBeath et al. (2004) cultured hMSCs on micropatterned materials with square shape ranging from 1000 to 10,000 μm^2^, and cells with different spreading area and differentiation commitments were obtained after 1 week. Osteogenesis was observed mostly on larger micropatterned substrates, whereas adipogenesis was found on smaller substrates, suggesting that cell spreading area induced by the micropatterned microenvironment can change the fate of MSCs. However, the spreading area varies with the shape. Thanks to the development of print technique, we can decouple these two parameters. Kilian et al. seeded individual MSCs on different rectangles but with constant area using the microcontact printing technique. Eventually, they found that the osteogenic differentiation of MSCs increased with the aspect ratio (1:1, 3:2, and 4:1), indicating that the shape of materials can independently affect the fate of MSCs (Kilian et al., 2010). Moreover, the development of manufacturing technology also makes it possible to reveal the effects of the cell–cell interaction. Tang et al. (2010) designed five different micromodel domains composed of microisland, allowing a single MSC to bind to each microisland, so that the average number of cell contacts in each micromodel domain is different. Finally, they observed that the osteogenesis and adipogenesis of MSCs are linearly related to the number of cell–cell contacts and gap junctions. In addition, in order to study the effects of alignment direction, Zhu et al. (2005) aligned and cultured MSC-derived osteoblast-like cells along the direction of nanogrooves in the scaffold. Finally, they pointed out that the cells implanted in the laser-treated nanogrooves had a higher proliferation rate than those in normal disk, suggesting that the nanoscale alignment along the longitudinal direction can promote the aligned formation of bone tissue. However, contrary to the above results, Jahani et al. (2012) showed that random nanofibrous scaffolds were more suitable for the growth of rat MSCs than aligned scaffolds and had higher proliferation. The reason may be that the random fibers contain more interconnected pores for circulation of nutrients. In addition, the significance of topography, such as the mussel-inspired nanostructures of functionalized 3D-printed bioceramic scaffolds developed by Li et al. (2019), could accelerate tissue regeneration by regulating the paracrine of adipose-derived MSCs. Based on the above researches, the topography of the matrix can affect the behavior of MSCs in different ways.

Because the topography are mainly physical stimulations, the underlying mechanism is related to the cytoskeletal contractility and the response of MSCs to mechanical forces. Kilian et al. (2010) demonstrated that topographical features of substrates increased MSCs’ actomyosin contractility so as to promote the osteogenesis of MSCs. At the same time, the increased cytoskeletal tension is related to the enhanced c-Jun N-terminal kinase (JNK) and the activation of extracellular-related kinase (ERK1/2), as well as the elevated wingless-type (Wnt) signaling (Kilian et al., 2010). Thanks to the advance in biomaterials preparation, we can gain insight into the underlying mechanism of topography and give full play to its role in MSC-based regenerative medicine.

### Pore Size and Porosity

The pore size and porosity have been valued by researchers for a long time. As early as 1971, Weber et al. (1971) pointed out that the structure of the porous medium is crucial. They stated that the interconnected pores with appropriate size, shape, and uniformity are essential for cell growth and adhesion. Since then, lots of experts have devoted themselves to studying the effects of pore characteristics on MSCs, in order to reveal its potential in the field of manufacturing biomaterials.

Although the optimal pore size of scaffolds varies with the biomaterials and cell type, the pore size and porosity are closely related to the cellular behavior of MSCs. Murphy et al. (2016) manufactured a series of collagen-glycosaminoglycan (CG) scaffolds with mean pore size ranging from 85 to 325 μm and found that the pore size exhibits a non-linear and bimodal effect on MSC adhesion. Specifically, MSCs adhered most to scaffolds with an average pore size of 325 μm, whereas another peak appeared at 120 μm. This phenomenon was due to the fact that the small pores have a larger surface area and the large pores have a higher ligand density. Similarly, Zhang et al. (2016) stated that the smaller pore size in the framework provided a larger surface area for cell adhesion. In addition, the pore size of biomaterials also changes the proliferation efficiency of MSCs. Zhang et al. fabricated a set of scaffolds with three different mean pore sizes (i.e., 215, 320, and 515 μm). Eventually, they claimed that the proliferation efficiency in the 515 μm scaffold was significantly lower than the other two groups. In other words, MSCs prefer to proliferate within the small pores of scaffolds. On the other hand, as demonstrated by O’Brien et al. (2007) the large pore size is superior to small pores in MSC migration or infiltration into the interior of the scaffold (Murphy et al., 2016). Therefore, when manufacturing the scaffold, we need to achieve a balance between utilizing large pores to improve cell migration and penetration and small pores to promote cell adhesion and proliferation. Meanwhile, the porosity also proved to be related to the behavior of MSCs. The highly porous patterns fabricated by electrospinning technology have been comprehensively assessed and proved to facilitate bone engineering (Ding et al., 2019). As we all know, the increase in porosity is beneficial to the nutrient diffusion and the waste removal within a certain range and achieves good proliferation of MSCs (Zhao et al., 2021). Kasten et al. (2008) implanted MSC-loaded β-tricalcium phosphate (TCP) scaffolds with different porosities (25, 65, and 75%) into SCID mice and found that the TCP 65 and TCP 75 with high porosity had higher ALP activity than the TCP 25 after 8 weeks. Obviously, the increase in porosity is conducive to the osteogenesis of MSCs *in vivo*. Like porosity, numerous studies have confirmed that the change of pore size can also guide the differentiation commitment of MSCs. Matsiko et al. (2015) cultured MSCs in Collagen-hyaluronic acid (CHyA) scaffolds with three different average pore sizes (94, 130, and 300 μm) and demonstrated that the maximum pore sizes (300 μm) significantly enhanced the expression of cartilage-forming genes and cartilage-like matrix deposition. In another research, Mygind et al. (2007) pointed out that the 200-μm pore hydroxyapatite scaffold exhibited faster osteogenic differentiation than the 500-μm pore scaffold, while the latter had a higher proliferation capability. It seems that there is an optimal pore range for MSC differentiation, and this range varies with the cell types and biomaterials. Pore size and porosity, as the specific form of pore structure, have a significant impact on the behavior of MSCs attached to biomaterials.

## Biochemical Cues

It has been many years since the biochemical modulation of stem cell growth and differentiation using small molecules and growth factors. However, due to the shortcomings of burst release and difficultly of long-term control or definition, biochemical factors have always been integrated into biomaterial-based scaffolds. Compared to biophysical cues, biochemical cues of biomaterials are easier to deliver. Besides growth factor and small bioactive molecules, genetic regulators have also been discussed in this section.

### Growth Factor and Derivatives

Some progress has been made in promoting MSC-based regeneration by adding growth factors and derivatives, such as EGF, VEGF, FGF, and TGF β. For example, soluble EFG can not only promote the proliferation of MSCs without compromising its pluripotent differentiation but also increase paracrine secretion to accelerate tissue regeneration (Tamama et al., 2010). However, conventional administration methods deliver growth factors and derivatives in soluble form, leading to complications such as hypotension and nephrotoxicity (Ferrara and Alitalo, 1999). So, combination of scaffold with growth factors would become an alternative and effective method and has made clear progress in areas such as bone regeneration of osteonecrosis (Zhu et al., 2020). Therefore, as a novel delivery method, the integration of growth factors into biomaterials can recapitulate a more suitable environment for MSCs at a physiologically relevant concentration and duration.

Fan et al. (2007) fabricated a scaffold covalently modified with epidermal growth factor (EGF) to control the release of EGF more precisely, which could increase MSCs spreading and adhesion through elevated ERK signaling and enhanced resistance to FasL-mediated cell death relative to saturating concentrations of soluble EGF. These advances can be attributed to the combination of EGF molecules with structures that bind and activate EGF receptors and the production of local high concentrations of EGF at the cell–matrix interface. As we all know, basic fibroblast growth factor (bFGF) can not only enhance the proliferation of MSCs but also promote the differentiation of MSCs in different directions (Rodrigues et al., 2010). In order to accurately mimic the release concentration of bFGF in injured ligament/tendon, Sahoo et al. (2010) incorporated bFGF-releasing PLGA fibers to the surface of the knitted silk scaffold, and the biological concentration of 6.5–13.5 pg/ml of bFGF in bioactive form was successfully recovered. Compared with bFGF (-) scaffold, MSCs in bFGF (+) scaffold exhibited higher viability (increased by 25%) during the whole culture process. Moreover, the gene expression level of ECM protein in the bFGF (+) scaffold such as type I and III collagens, fibronectin, and the deposition of soluble collagen were significantly increased. These results indicated that the released bFGF has broad application prospects in repairing tendon and ligament injury by promoting proliferation and tenogenic differentiation. On the other hand, vascularization of grafts plays a very important role in tissue repair and vascular endothelial growth factor (VEGF) is an essential growth factor in regular angiogenesis (Davies et al., 2008). Khojasteh et al. (2016) fabricated a porous scaffold with VEGF controlled release and proved that the proliferation and attachment of MSCs were significantly increased than those without VEGF. Meanwhile, they found that the gene expression of COL I and RUNX2 for osteogenesis and that of vWF and VEGFR2 for angiogenesis were statistically increased, demonstrating that VEGF not only promoted angiogenesis but also played an important role in bone repair. Besides, TGFβ is also a known superfamily that can affect the chondrogenic differentiation and matrix deposition of MSCs *in vivo* (Rodrigues et al., 2010). Therefore, lots of studies have integrated TGFβ into the scaffold to explore its prospects in cartilage repair. Re’em et al. (2012) developed a scaffold with TGFβ sustained release, and the chondrocytes with deposited type II collagen were only found in the TGFβ bound constructs compared to the naked one. Therefore, the use of biomaterials to deliver growth factors to regulate MSCs behavior provides a viable means for tissue regeneration.

Because MSCs are regulated by a variety of regulatory factors *in vivo*, the combination of multiple biological factors on a biomaterial or delivery system is also a promising approach. Moreover, the synergistic effect on target cells is the characteristic of integrating multiple factors into one system. For example, Simmons et al. (2004) fabricated a dual growth factor delivery alginate hydrogel scaffold in which bone morphogenetic protein-2 (BMP2) and transforming growth factor-β3 (TGF-β3) are incorporated. The scaffold supported the simultaneous release of important growth factors during osteogenesis from MSCs, and the dual delivery system showed more efficient and more effective tissue regeneration *in vivo* compared to the individual delivery. In another research, insulin-like growth factor-1 (IGF-1) and TGF-β1 were loaded together in gelatin microparticles (Park et al., 2009). Then, the gelatin microparticles and rabbit marrow MSCs were assembled into an injectable hydrogel. Over the culture time *in vitro*, TGF-β1 was gradually found to accelerate chondrogenic differentiation of MSCs, while IGF-1 promoted cell aggregation. The incorporation of the two growth factors showed a synergistic effect on chondrogenesis of MSCs, which provided a great potential for cartilage regeneration and repair. The above studies showed the advantages of multiple factor delivery biomaterials; thus, how to construct a novel system to provide appropriate combination of multiple growth factors or simulants is an inevitable trend in future researches and even clinical applications (Richardson et al., 2001).

However, due to the immunogenicity and short half-life, the integration of large protein growth factor into polymer scaffolds has certain limitations (Cai et al., 2014). Growth factor derivatives and peptides are short peptide sequences that mimic the receptor-binding or functional domains of growth factors (Liu et al., 2012). These short peptides can bind to corresponding receptors to activate intracellular pathways to achieve the effect of growth factors, while avoiding the obstacles of large proteins. Currently, it has been reported that short peptides such as QK, KLT, PRG, and the RGD can achieve this goal, and RGD sequence has been extensively tethered to biomaterials to regulate MSC behaviors for tissue repair (Liu et al., 2012; Lam and Segura, 2013; Cai et al., 2014; Rao et al., 2020). Yang et al. (2005) covalently incorporated different dosage adhesion peptides Arg-Gly-Asp (RGD) into hydrogel and found that the expression of bone-related markers ALP and OCN was significant higher in RGD-conjugated hydrogel than the control. Furthermore, the level of gene expression was positively correlated with the concentration of RGD. Consistent with Yang, the RGD peptide was bound to the surface of the scaffold by Qu et al. (2010). Compared with the unmodified scaffold, more MSCs adhered to RGD scaffolds after 4 h of culture. Moreover, after 14 days of culture, the RGD-modified scaffolds significantly promoted the osteogenesis of MSCs. These studies show that the growth factor derivatives and short peptide factor have good prospects and operability in regulating the behavior of MSCs.

### Small Bioactive Molecules

Small bioactive molecules, such as nitric oxide, oxygen, and metallic ions, also have significant effects on MSCs behavior. Since 1977, Arnold et al. (1977) discovered that NO could participate in various physiological processes by activating the cyclic guanosine monophosphate (cGMP), and it has been intensively studied in cardiovascular homeostasis, tissue repair, and immunomodulation. Yao and his group cultured adipose-derived MSCs in a hydrogel that can release NO molecule continuously and transplanted the hydrogel into murine myocardial infarction (MI) models, which achieved positive therapeutic effects (Yao et al., 2015). It has been widely confirmed that the protective effect of MSCs on MI is mainly achieved through the pro-angiogenic cytokines it secreted (Berardi et al., 2011). Similarly, Yao and co-workers discovered that NO hydrogel remarkably enhanced the paracrine and the VEGF secretion of MSCs. Finally, these cytokines improved heart function by promoting vascularization and reducing ventricular remodeling. In another approach, the effects of NO on other aspects of MSCs was observed by Xing et al. (2013). He fabricated a gelatin hydrogel that can release nitric oxide at a physiological concentration, and lower attachment and proliferation efficiency of MSCs were obtained on the NO hydrogel compared to the control after incubating for 72 h. Therefore, utilizing NO to modify the scaffold can improve antithrombotic ability by reducing cell adhesion and proliferation, which can be used as a coating material for repairing vascular injury. However, the instability of NO and its oxidation potential to the toxic nitrogen dioxide molecule is still the barrier to extremely exploit the therapeutic effects of NO (Xiao et al., 2017). So, the development of a more secure and stable delivery system becomes a breakthrough of the next-generation NO-based biomaterials.

Oxygen level is also a critical regulator of stem cell behavior. At present, MSCs are usually cultured in an incubator under an oxygen level of 20% pO_2_, whereas the residing niche MSCs are in low oxygen tension (1–7% pO_2_) (D’Ippolito et al., 2006). Therefore, more and more studies have examined the effects of oxygen level on MSCs. It is already known that low oxygen tension (hypoxia) can not only maintain the stemness of stem cells but also influence their proliferation and differentiation (Mohyeldin et al., 2010). Zhou et al. (2014) seeded bone marrow-derived MSCs into scaffold and cultured in different oxygen tension, and the MSCs under hypoxia (5% pO_2_) exhibited a higher proliferation response compared with the others cultured under normoxic conditions (20% pO_2_) on day 4 and 10. Meanwhile, higher levels of Runx2, Bmp2, BMP, and VEGF were observed in the hypoxia scaffold relative to the normoxic scaffold, suggesting that the hypoxia is conducive to the osteogenesis and angiogenesis of MSCs. In addition, Tong et al. (2016) demonstrated that hypoxic pretreated MSC-containing biomimetic scaffold observably accelerated wound healing in diabetic rat ulcer. The reason is that hypoxia pretreatment can enhance the secretion of proangiogenic factors and angiogenesis of MSCs.

As cofactors of enzymes, various metal ions are widely involved in tissue homeostasis and participated in lots of chain reactions related to cell signaling pathways (Gérard et al., 2010). In recent years, more and more evidences showed that metallic ions also play an important role in the field of regenerative medicine by regulating MSCs behavior. Meanwhile, the integration of metal ions into bioactive scaffolds generated a dual function for matrix and enhanced its therapeutic effect (Mouriño et al., 2012). The main reasons were due to the fact that these ions released from the scaffold can stimulate various processes, including proliferation, attachment, and differentiation. For example, the enhanced osteogenic differentiation was observed on a silicon-releasable scaffold, and the silicon species in the scaffold was regarded to promote the alkaline phosphatase (ALP) and osteogenesis (Obata and Kasuga, 2009). The silver nanoparticles were also found to promote the proliferation and osteogenesis of MSCs *in vitro*, and the improved bone fracture healing was obtained through a novel collagen reinforced by silver nanoparticles (Zhang et al., 2015). Therefore, a comprehensive understanding of the effects of metal particles on MSC behaviors is necessary for the development of metal ion-integrated biomaterials. Table 1 summarizes the effects of different metallic ions on the behavior of MSCs. However, achieving sustained release of metal ions under suitable concentration and without systemic toxicity remains difficult as the complexity of manufacturing process is still a challenge for these strategies based on controlled metal release.

**TABLE 1:** Effects of metallic ions on MSCs.

Ion	Ionic form	Experimental trial	Effects on behaviors	References
Silicon	Glass microspheres (BGMs) Silicon-releasable scaffold Composite hydrogel	*In vitro In vitro In vitro/vivo*	Enhanced the attachment and proliferation of human MSCs Induce and enhance the osteogenic differentiation of MSCs Angiogenesis and adipogenesis	Lei et al., 2011 Obata and Kasuga, 2009 Wang et al., 2018
Calcium	Calcium phosphate composition	*In vitro*	High mobility of focal adhesion, osteogenesis without induce medium	Muller et al., 2008
Cobalt	Cobalt chloride composition Cobalt chloride composition Cobalt chloride solution	*In vitro In vitro In vitro*	Increase the chondrogenic markers such as SOX9, COL2A1, VCAN, ACAN Induce neuronal differentiation Enhance migration of MSCs	Teti et al., 2018 Jeon et al., 2014 Yu et al., 2013
Copper	Copper–histidine complex	*In vitro*	Modify differentiation and proliferation by different concentrations	Rodriguez et al., 2002
Zinc	Zinc-added bioactive glass	*In vitro*	Induce growth and osteogenic differentiation of MSCs	Oh et al., 2011
Vanadium	Vanadium-loaded collagen scaffold Vanadium-released scaffold	*In vitro In vitro*/*vivo*	Adhesion, growth, differentiation Endochondral ossification and angiogenesis *in vivo*.	Cortizo et al., 2016 Schussler et al., 2017
Strontium	Strontium-ranelate solution Strontium-collagen scaffold	*In vitro In vitro*/*vivo*	Osteogenic induction of MSCs at appropriate concentration Enhance osteogenic differentiation and bone formation	Sila-Asna et al., 2007 Yang et al., 2011
Iron	Iron oxide nanoparticles	*In vitro*	Accelerate cell cycle progression, promote cell growth	Huang et al., 2009
Magnesium	Magnesium-extract solution Magnesium alloys extracts	*In vitro In vitro*	Cell proliferation, osteoblastic differentiation Enhance proliferation and osteogenic differentiation	Luthringer and Willumeit-Romer, 2016 Li et al., 2014
Silver	Silver nanoparticles Silver nanoparticles	*In vitro In vitro/vivo*	Induce MSCs activation at appropriate concentration Promote the proliferation and osteogenesis of MSCs	Greulich et al., 2009 Zhang et al., 2015

### Genetic Regulators

Recently, great achievement has been made in regulating the behaviors of cells by integrating genetic regulatory factors into biomaterials. For example, the improved cell proliferation, enhanced osteogenesis, and high-quality healing of large-scale bone defects were obtained by a type of multi-functional scaffold containing phBMP-4 through controlled and sustained gene expression (Cui et al., 2020). The successful delivery of genes based on viral and non-viral means plays a great role in genetic-based tissue engineering (Huard et al., 2003; Giatsidis et al., 2013; Mellott et al., 2013). Currently, non-viral vectors are more preferred for gene therapy because viral vectors are immunogenic and carry the risk of infection and cytotoxicity (Giatsidis et al., 2013). Moreover, the physical non-viral transfection methods such as electroporation significantly improved the transfection efficiency to control the cellular behavior during tissue regeneration (Mellott et al., 2013). Here, we review the effects of genetic regulatory factors (such as complementary DNA and small interfering RNA) on MSCs.

Complementary DNA (cDNA) is a nucleic acid sequence that can encode specific proteins by reverse transcription in transfected cells. It has been reported that various specific proteins including VEGF (Huang et al., 2005), EGF (You and Nam, 2013), bFGF (Yau et al., 2007), BMP (Meinel et al., 2006; Wegman et al., 2011), and hepatocyte growth factors (HGFs) (Lu et al., 2013) can guide behaviors of MSCs through transfecting corresponding cDNA. Wegman et al. (2011) achieved osteogenic differentiation of MSCs *in vitro* and *in vivo* through prolonging expression of BMP-2 through plasmid DNA-based gene therapy. They incorporated the BMP-2 cDNA into an alginate hydrogel, and seeded MSCs into the hydrogel before implanting to naked mice. In the end, the continuous expression of BMP-2 protein significantly promoted the osteogenic differentiation of MSCs, which was also verified by the increased expression of ALP and the deposition of collagen I and osteocalcin. In another study, Moon et al. (2014) fabricated modified polyethyleneimine (PEI) conjugates to deliver VEGF cDNA and demonstrated that VEGF-MSCs enhanced capillary formation in the infarcted area and attenuated ventricular remodeling in the model of MI.

Small interfering RNA is a small double-stranded RNA sequence (21–23 nucleotides) that can silence and knock down target genes by complementary binding to the corresponding mRNA sequence. Therefore, siRNA may provide another effective intervention to guide stem cell behavior in regenerative medicine applications (Benoit and Boutin, 2012). Jia et al. (2014) integrated two small interfering RNAs into the chitosan sponge, one targeting casein kinase 2 interaction protein 1 (siCkip-1) and another targeting soluble VEGF receptor 1 (siFLT-1), and osteogenesis and angiogenesis were observed *in vitro* and *in vivo* after co-culturing with MSCs. Moreover, they pointed out that the osteogenesis may be due to the targeted knockdown of Ckip-1 that markedly activates the signaling pathways related to bone morphogenetic proteins. At the same time, silencing soluble VEGF receptor 1 gene eventually upregulated the release of VEGF, which promoted the angiogenesis of MSCs. Similarly, we can also see the same phenomenon in the research of Nagai (Nagane et al., 2010). The author transformed the osteogenesis of MSCs to adipogenesis through culturing MSCs with the siRNA-containing cationic dextran. The reason was that the siRNA here can knock down the activity of transcription coactivator PDZ-binding motif (TAZ), thus significantly promoting the osteogenesis of MSCs rather than adipogenesis.

## Potential of Combining Biochemical and Biophysical Cues

Because the biophysical and biochemical signals exist simultaneously and cooperate together *in vivo*, combining their effects *in vitro* as an alternative solution has become an increasingly clear and affirmative topic in the field of cellular therapy and regenerative medicine. More importantly, several groups have proved the feasibility of combining the effects of biophysical and biochemical signals *in vitro*. For example, a novel osteogenic polypeptide hydrogel (GelMA-c-OGP) in which GelMA enabled the formation of hydrogel with mechanical properties, combined with osteogenic growth peptides (OGP) through co-cross-linking that continuously release during the bone defect healing period, was created by Qiao et al. (2020). Finally, the interaction of the two parameters promoted the bone formation procedure of osteogenic precursor cells *in vitro*, and more collagen fibers were observed to connect with cortical bones after implantation. In another study, an *in vitro* model system combining biochemical and biophysical factors was found to be more effective on cardiomyocyte differentiation from rat bone marrow-MSCs (BM-MSCs) than any single factor alone (Ge et al., 2009).

**FIGURE 2:**
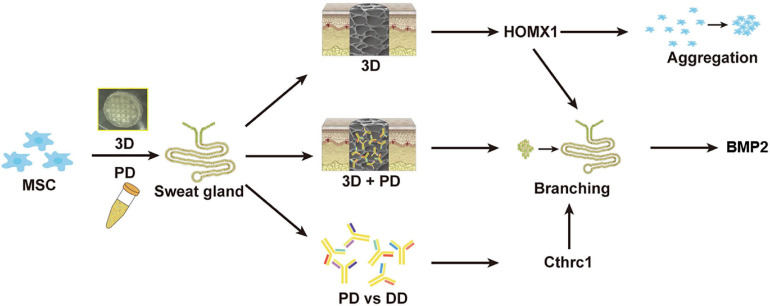
The graphic illustration of 3D bioprinted matrix-directed MSC differentiation. CTHRC1 is the main biochemical cue during SG development and structural cues upregulated the expression of hmox1, synergistically initiating branching morphogenesis of SG.

In recent years, 3D bioprinting offers a promising and alternative platform to fabricate tissue-specific constructs for tissue repair and regeneration. These 3D functional constructs are regarded as the most biomimetic module due to the fact that they can provide both biochemical cues and biophysical signals to regulate cell–cell and cell–ECM interaction. In our previous work, by 3D bioprinted specific sweat gland (SG) matrix, we can differentiate MSCs into functional SGs by combination of biochemical and structural cues (Figure 2). The 3D printed SG-like matrix provided a novel strategy to combine chemical factor, especially the collagen triple helix repeat containing 1 (CTHRC1), and 3D structural factor (Yao et al., 2020). These two cues synergistically directed MSCs’ commitments into the glandular lineage and functional SG recovery *in vitro* and vivo. In addition, 3D bioprinting of tissue-specific decellularized extracellular matrix (dECM) bioink can provide a complex site-specific combination of biochemical and mechanical cues, which have been hypothesized to prove their potential application in tissue engineering.

In addition to the synergistic effects, some researches have revealed the competitive relationship between the biophysical cues and the biochemical cues. For example, human bone marrow-derived MSCs were cultured on the nanostructured surface in differentiation media by McMurray et al. (2011). Compared with the cells cultured in the inductive medium alone, one interesting phenomenon was that the cells on the surface of nano-patterned topography showed a higher degree of stemness maintenance and lower differentiation levels. The reason is probably that biophysical factors have a longer duration and lifetime, which could even convert the effects of biochemical stimulation (Ahsan and Nerem, 2010). However, it is not clear whether these two aspects will conflict with each other, as well as the extent and the underlying mechanism. Therefore, distinguishing the internal effects when biophysical and biochemical cues are used in combination is essential.

## Conclusion and Future Prospects

The key point of MSC-based therapy is to maintain the viability of MSCs and accurately guide their fate and functionalization at the injury site. However, once MSCs are separated from their niches, their phenotype, functionality, and viability can easily be distorted. Therefore, it is necessary to create highly mimicking native-room biomaterials or substrates for MSCs to maintain their properties and exhibit therapeutic effects. Here, the biophysical and biochemical factors that determine the behaviors of MSCs have been discussed. These different cues and different protocols utilized in a variety of studies have led to difficulties in obtaining clear conclusions about the effects on regulating the proliferation, adhesion, and differentiation of MSCs and ultimately affect the function of the target tissue. Therefore, a comprehensive and systematic understanding of these signals is essential for the synthesis of more advanced artificial biomaterials and tissue engineering. Table 2 summarizes the factors that fall into some categories.

**TABLE 2:** Effects of biomaterials on MSCs.

Category		Effects on behaviors	References
Biophysical	Stiffness	Proliferation	Whitehead et al., 2018
		Spreading	Engler et al., 2006; Wingate et al., 2012
		Differentiation	Du et al., 2011; Shih et al., 2011; Higuchi et al., 2013; Sun et al., 2018
	Stress relaxation	Proliferation	Cameron et al., 2011; Chaudhuri et al., 2016
		Spreading	Cameron et al., 2011; Chaudhuri et al., 2016
		Differentiation	Cameron et al., 2011, 2014; Darnell et al., 2017
	Topography	Proliferation	Zhu et al., 2005; Jahani et al., 2012
		Spreading	McBeath et al., 2004
		Differentiation	McBeath et al., 2004; Kilian et al., 2010; Tang et al., 2010
		Paracrine	Li et al., 2019
	Pore size and porosity	Proliferation	Mygind et al., 2007; Zhang et al., 2016; Zhao et al., 2021
		Differentiation	Mygind et al., 2007; Kasten et al., 2008; Matsiko et al., 2015
		Adhesion	Murphy et al., 2016; Zhang et al., 2016
		Migration	O’Brien et al., 2007
Biochemical	Growth factor and derivatives	Proliferation	Rodrigues et al., 2010; Sahoo et al., 2010; Khojasteh et al., 2016
		Differentiation	Simmons et al., 2004; Park et al., 2009; Rodrigues et al., 2010; Sahoo et al., 2010; Re’em et al., 2012; Khojasteh et al., 2016; Rao et al., 2020; Zhu et al., 2020
		Paracrine	Tamama et al., 2010
		Spreading	Fan et al., 2007; Lam and Segura, 2013
		Adhesion	Fan et al., 2007; Qu et al., 2010; Khojasteh et al., 2016
	Small bioactive molecules (nitric oxide)	Immunomodulation	Yao et al., 2015
		Paracrine	Berardi et al., 2011
		Differentiation	Berardi et al., 2011
		Adhesion	Xing et al., 2013
		Proliferation	Xing et al., 2013
	Small bioactive molecules (oxygen level)	Proliferation	Zhou et al., 2014
		Differentiation	Zhou et al., 2014; Tong et al., 2016
		Paracrine	Tong et al., 2016
	Small bioactive molecules (metallic ions)	Table 1	
	Genetic regulators (cDNA)	Differentiation	Huang et al., 2005; Meinel et al., 2006; Yau et al., 2007; Lu et al., 2013
		Proliferation	You and Nam, 2013
		Migration	You and Nam, 2013
		Paracrine	Moon et al., 2014
	Genetic regulators (siDNA)	Differentiation	Nagane et al., 2010; Jia et al., 2014
Combined strategy	Mechanical property and polypeptides	Differentiation	Bauer et al., 2017
	Strain and biochemical extract factors	Differentiation	Ge et al., 2009
	3D microenvironment and biochemical extract factors	Differentiation	Yao et al., 2020

Recent advances in the design and manufacture of biomaterials with tailoring parameter not only provide a versatile toolbox for bioengineering [e.g., embedded 3D bioprinting (de Melo et al., 2019), co-fabrication (Jodat et al., 2020), and stereolithography (Kumar and Kim, 2020)] but also present a feasible approach for subsequent research. However, we believe that the development of the biomaterial for regenerative medicine needs to be combined with multidisciplinary research progress, and the biophysical and biochemical factors used to regulate MSC behaviors must be precisely integrated and adjusted according to the needs of the repair area. Just like Yasamin et al., they combined multi-material 3D bioprinting with electronic platform technology to synthesize a hybrid device that can not only reconstruct the mechanical structure of nasal cartilage but also sense odor (Jodat et al., 2020). The integration of the aforementioned multidisciplinary research can be a potential avenue toward achieving functional nasal regeneration and organ transplant. Therefore, we believe that the next-generation biomaterials with accurately integrated induction cues for MSCs will open a new window for perfect regeneration and simultaneous repair.

## Author Contributions

SH contributed to the conception of the study and edited the manuscript. JL and YL wrote the sections of the manuscript. All authors read and approved the submitted version.

## Conflict of Interest

The authors declare that the research was conducted in the absence of any commercial or financial relationships that could be construed as a potential conflict of interest.
